# Enhancing medical students’ self-efficacy toward national competency standards: a student-led training model in a lower-middle-income country

**DOI:** 10.1186/s12909-025-08335-9

**Published:** 2025-12-08

**Authors:** Yusof Mohamed Omar, Basma Kamel, Shaimaa Nasr, Mohamed Ibrahim Morshed, Tasneem Deibes, Mohamed Hossam Ghazy, Rasha Samir Shemies

**Affiliations:** 1 Faculty of Medicine, Mansoura University, Mansoura, Egypt; 2 Mansoura Students’ Scientific Association, Faculty of Medicine, Mansoura University, Mansoura, Egypt; 3 Medical Program, Mansoura University Hospitals, Mansoura, Egypt; 4 Mansoura Nephrology and Dialysis Unit, Mansoura University Hospitals, Mansoura, Egypt

**Keywords:** Self-efficacy, Medical students, Student-led programs, Competency-based education, Egypt

## Abstract

**Background:**

Following a national reform, Egyptian medical schools adopted the National Academic Reference Standards (NARS) in a transition toward competency-based education. However, preparing graduates to meet these standards remains challenging amid rapidly increasing student numbers and limited resources. This study evaluated the impact of the student-led Shouman Summer Training 2023/2024 (SST24) on trainees’ self-efficacy toward the NARS competencies.

**Methods:**

SST24 took place in August 2024 at Mansoura University Hospital’s departments across two 14-day waves. The program enrolled 652 trainees across 31 clinical departments, and combined hands-on bedside teaching, supervised procedures, and two supplementary workshops aligned with the NARS competencies. A pre–post observational design was used to assess changes in three outcomes: 1) self-efficacy toward NARS competencies measured with a 5-point self-report questionnaire; 2) clinical empathy, using the Jefferson Scale of Empathy (JSE-S); and 3) clinical knowledge, assessed with department-specific quizzes. Pre-post changes were analyzed using paired t-tests, mean differences (MD), and Cohen’s d.

**Results:**

Of 652 trainees, 535 met inclusion criteria for the final analysis (82%). The program led to significant overall gains in self-efficacy toward NARS competencies (*p* < .001), but the outcomes varied depending on the training environment. Trainees in ‘People-Oriented’ departments, which emphasized patient interaction, reported higher gains in identifying health risks and adopting infection control measures compared to their peers in technical settings (MD = 0.85; d = 0.93 vs. MD = 0.49; d = 0.38). Conversely, trainees in ‘Technology- and Procedure-Oriented’ departments reported greater self-efficacy gains in adopting patient safety measures (MD = 1.64 vs. 1.31) and adhering to professional standards (MD = 0.72 vs. 0.58). However, the intensive focus on technical skills in these departments was associated with a modest but significant decline in empathy (mean JSE-S score change = -2.34). Clinical knowledge scores also improved significantly, with mean correct answers increasing by 15.8 and 14.1 percentage points in technology- and people-oriented departments, respectively (*p* < .001).

**Conclusions:**

SST24 enhanced trainees’ self-efficacy towards NARS competencies, as well as department-specific knowledge, demonstrating a scalable, student-led model for resource-limited settings.

**Supplementary Information:**

The online version contains supplementary material available at 10.1186/s12909-025-08335-9.

## Introduction

Graduating competent healthcare providers requires a robust educational framework that bridges the gap between theoretical knowledge and practical application. Recognizing this necessity, medical education worldwide has shifted in recent years from traditional, time-bound, teacher-centered models to competency-based frameworks designed to ensure graduates possess the skills and behaviors needed for independent practice [1, 2]. In 2018, Egypt formalized the transition to competency-based medical education (CBME) by implementing the National Academic Reference Standards (NARS) [3]. The NARS framework delineates core professional attributes—such as professionalism— and specifies the corresponding competencies expected of graduates, including adherence to ethical standards and the maintenance of patient confidentiality. These standards aim to prepare graduates to meet the demands of modern healthcare while promoting continuous professional development. However, various challenges have impeded the full implementation of CBME and, consequently, the achievement of NARS among medical students in Egypt [4, 5].

Since the 2017 reform, however, medical education in Egypt has experienced rapid expansion, with growth in enrollment and institutions exceeding the pace of infrastructure and faculty development. The number of medical students enrolled in public universities has nearly doubled, while the number of medical schools increased from 29 in 2017 to 51 as of 2025 [6]. Much of this growth stems from the rapid establishment of private and national (Al-Ahlia) universities, many of which lack their own teaching hospitals, despite now enrolling approximately 23.4% of all medical students [6]. Consequently, already overburdened public university hospitals must accommodate not only their expanding student cohorts but also large numbers from these newer schools. The resulting strain on clinical training capacity is further compounded by inadequate faculty-to-student ratios, limited funding, and outdated infrastructure [4, 5, 7]. Collectively, these challenges hinder the effective implementation of CBME and leave many students ill-prepared for clinical practice. To mitigate this gap, student-led initiatives, such as student-run clinics [8] and clinical learning environments [9], have emerged as practical supplements in similarly strained educational settings, creating alternative supervised training opportunities and helping redistribute the clinical teaching burden from overstretched faculty [10].

Exemplifying such student-led initiatives is the Shouman Summer Training (SST) program, conducted for over a decade at Mansoura University Hospitals and accommodating approximately 600 medical students annually. Organized each summer by the Mansoura Students’ Scientific Association (MSSA) under faculty supervision, SST provides trainees with the opportunity to develop clinical skills and empathy in a supportive environment free from the typical stressors of the academic year. The program allows students to explore various medical departments they may later pursue as specialties, helping shape their professional identity and set realistic expectations for post-graduation clinical practice, an issue of growing concern among medical educators [11, 12]. What makes SST particularly unique is that it is primarily student-organized and incorporates peer teaching, collaborative learning, and reflective practices, all within a resource-constrained setting. Drawing on the principles of experiential learning, SST aims to foster development across multiple learning domains, including the cognitive (clinical knowledge), psychomotor (procedural skills), and affective (attitudes and values) domains, particularly empathy, teamwork, and professionalism [13–15].

Amid the systemic challenges facing medical education in Egypt, SST provides a practical approach to addressing persistent gaps in clinical training. By leveraging available hospital departments and teaching staff during the summer vacation, SST delivers structured clinical exposure without requiring substantial new infrastructure. The program also relies on student organizers and supervisors to coordinate activities and facilitate peer learning, thereby reducing additional administrative and supervisory demands on faculty. In this context, the present study evaluates the effectiveness of the 2023/2024 SST program (SST24) by assessing its impact on trainees’ self-efficacy toward national competency standards. In doing so, it contributes to the growing body of literature on implementing CBME in lower-middle-income countries (LMICs), highlighting a relatively cost-efficient, student-driven model that enhances trainees’ confidence in core competency domains while easing institutional pressure.

## Methods

### Study design and period

This study utilized a pre-post observational design to evaluate the contributions of SST24 to trainees’ self-efficacy toward NARS competencies. SST24 consisted of two waves. The first wave was conducted from the 3rd to the 15th of August, and the second wave from the 17th to the 29th of August, lasting two weeks each. The study proposal was approved by the Institutional Research Board (IRB) of the Faculty of Medicine, Mansoura University (R.24.08.2756).

### Study setting

SST24 took place in 31 departments at Mansoura University Hospitals. This included medical departments such as Cardiology, Neurology, and Endocrinology, and surgical departments such as Cardio-thoracic Surgery, Orthopedic Surgery, and Neurosurgery. Participants were distributed across the departments based on department capacity and ranked student preferences obtained during their application process to align with individual learning interests and optimize an authentic workplace-based learning environment. A detailed list of departments, along with trainee distribution and average training hours, is provided in the supplementary material (Supplementary Material 1).

### Study participants

The study participants included second-, third-, and fourth-year medical students from Mansoura University Faculty of Medicine who were accepted into SST24 following a competitive application and selection process. First-year students weren’t eligible to apply due to their limited clinical knowledge, which would prevent them from fully benefiting from the training. Fifth-year students were also ineligible, as the timing of their internships shortly after the training period would leave them with limited opportunity to reflect on their experiences.

### Program description

**Fig. 1:**
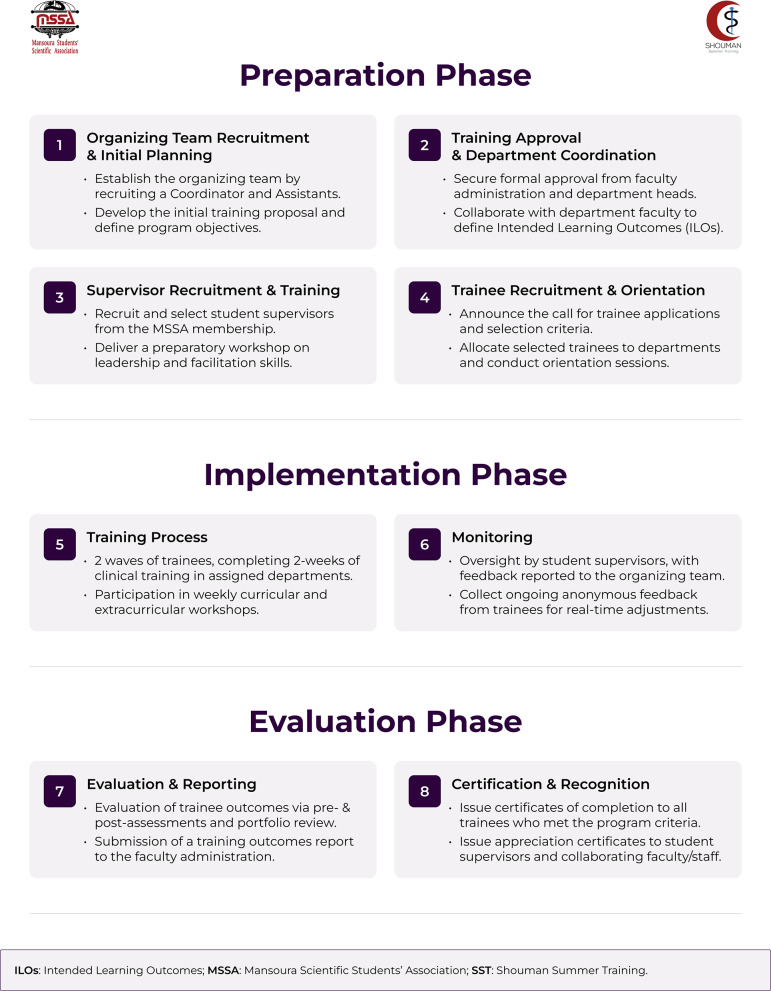
Organizational framework and workflow of the SST24 program

SST24 was structured into three primary stages: a preparation phase, an implementation phase, and an evaluation phase. The preparation phase focused on establishing the program's foundation through recruiting the organizing team and training supervisors, securing departmental approvals and developing intended learning outcomes (ILOs), and finally, recruiting trainees and conducting a pre-training orientation. The implementation phase included the two waves of 14-day clinical training, which were designed to align with Kolb’s experiential learning cycle [16]. The training concluded with a final evaluation phase that involved pre- and post-assessment analysis, validation of trainee portfolios, and reporting to the faculty administration. An overview of the SST24 structure is shown in Fig. 1. SST24 was funded through a nominal administrative fee paid by trainees, covering logbooks, identification, and participation certificates. Clinical training took place within Mansoura University teaching hospital departments where departmental supervisors, assigned by department heads after voluntarily agreeing to participate, guided trainees. Curricular workshops were held in faculty buildings, provided at no cost by the administration, and delivered by faculty teaching staff on a voluntary basis. Most organizing and program supervisory roles were carried out by MSSA student volunteers.

### Organizing team and training supervisors

Two months before the training, a call for applications was issued to all MSSA members to form the organizing team, which included a lead coordinator, one curricular coordinator assistant, one extracurricular coordinator assistant, two publications assistants, two monitoring and evaluation assistants, and two general assistants. The lead coordinator oversaw program design, implementation, and monitoring in collaboration with the assistants. The curricular and extracurricular coordinator assistants managed workshops and training activities, the publications assistants handled marketing, documentation, and promotion, the monitoring and evaluation assistants oversaw data collection, attendance tracking, and analysis of trainee feedback, while the general assistants supported the coordinator in preparation, supervisor follow-up, and final report preparation. Candidates were selected based on their motivation, proposed plans, relevant experience, and availability. The organizing team was tasked with drafting the training proposal for review and approval by the faculty administration. Once approved, they secured necessary approvals from department heads and collaborated with the department’s teaching staff to develop appropriate intended learning outcomes and assessments for the training.

Once training preparations were complete, a call for training supervisors was issued to MSSA members. Supervisors were selected based on their motivation, relevant experience, and availability. Once selected, each supervisor was assigned to one of the two training waves according to the needs and schedule of the program. Their responsibilities included facilitating communication between trainees, department teaching staff, and the organizing team, ensuring smooth execution of the training, and collecting and reporting feedback throughout the program to support continuous improvement, and consistency with Kolb’s learning cycle [16]. To prepare them for their roles, a structured 8-h workshop was conducted prior to the program’s start, focusing on leadership, communication, and facilitation skills, essential for supporting experiential learning processes [17].

### Call for trainees’ applications and pre-training orientation

A call for trainee applications was announced on all MSSA social media platforms, targeting Mansoura University medical students and providing details about the training and outlining the acceptance criteria. Trainees were selected based on several factors, including their motivation, academic year, and prior participation in the training. Preference was given to students in earlier academic years, as they had more time to reflect on and apply their training experiences during their remaining study years. Priority was also given to students who had not previously participated, ensuring a broader pool of participants could benefit from the program. All applications were reviewed blindly by two organizing team members and approved by the MSSA executive board. Accepted trainees were then allocated to departments based on the list of preferences they submitted in their applications, the score of their application, and the capacity of each department.

An orientation session was held for all trainees before the start of their respective waves. The session introduced the program objectives, hospital protocols, and the communication chain within the program, outlining the roles of trainees, supervisors, department staff, and the organizing team. It also outlined the study purpose and the requirements for receiving a training certificate, which included completing both pre- and post-assessments and the trainee portfolio, as well as attending at least 70% of the training hours and participating in one curricular and one extracurricular workshop.

### Training process

SST24's design incorporated experiential learning principles, emphasizing situated learning, active participation, and guided reflection. These principles translated into practice through following Kolb’s four-stage cyclical model of knowledge development, where the trainees moved through concrete experience, reflection, abstract conceptualization, and active experimentation. Aligned with the constructivist approach, the training prioritized authentic, practice-based experiences, social interaction, and reflective learning as core components of medical education [15, 16].

Under direct supervision, trainees engaged in clinical environments across surgical and medical departments, where departmental staff played a central teaching role. They participated in Concrete Experience through supervised activities such as history taking, physical examinations, basic surgical skills, and diagnostic test interpretation, while simultaneously engaging in Reflective Observation by maintaining structured portfolios documenting at least three clinical cases per day, including brief summaries, personal reflections, and areas for improvement. Throughout the training, trainees applied Active Experimentation by trying new approaches, such as enhanced communication strategies or examination techniques, to achieve the ILOs of their assigned departments, with supervisors providing immediate formative feedback.

### Supplementary workshops

To further support Kolb’s Abstract Conceptualization, two weekly workshops were conducted covering topics aligned with NARS competencies. The curricular workshop, delivered by faculty, focused on enhancing trainees’ clinical skills and knowledge, including history taking, managing emergency situations, and ethical aspects of medicine. The extracurricular workshop, facilitated by MSSA student trainers, emphasized inter-professional teamwork and experiential learning through simulation, role-play, and peer teaching, providing opportunities for trainees to reflect on and integrate their practical experiences.

### Training monitoring and evaluation

To ensure the quality and continuous improvement of the training, a multi-level monitoring and evaluation system was implemented. Throughout the training, trainees provided regular feedback to their assigned supervisors, who in turn relayed this input to the organizing team. In addition to this direct feedback loop, anonymous feedback forms were available for trainees to report any challenges, suggestions, or concerns they encountered during the training.

Trainee performance and program outcomes were assessed using a structured assessment tool specifically developed for the training. The tool was administered at both the beginning and end of the training to measure changes in trainees’ self-efficacy toward NARS competencies, empathy, and knowledge.

To receive the training certification, trainees were required to adhere to the training’s code of conduct, attend at least 70% of the training hours, participate in one curricular and one extracurricular workshop, complete both the pre- and post-assessments, and finalize their portfolio by the end of the training.

### Assessment tool and data collection

To evaluate the outcomes of SST24 training, we utilized an online pre- and post-assessment tool. Our tool was co-developed by the SST24’s organizing team and the faculty departments' teaching staff through consensus-building and iterative feedback cycles ensuring both trainee performance and program impact assessment. It comprised four sections, with the first section collecting anonymized demographic data, including participants’ age, sex, and academic year.

The second section evaluated SST24’s contribution to the NARS competencies. However, rather than measuring demonstrated competence directly, this section was designed to assess trainees’ self-efficacy—i.e., their belief in their capability to perform tasks delineated in the NARS [18]. This approach was chosen because direct assessment of competence through objective, externally rated measures was not feasible given the large number of trainees, the short and varied clinical rotations, and the limited human resources. Moreover, self-efficacy is a well-established precursor to, though not a direct measure of, competence [19]. Collaboratively developed by the training’s organizing team and the faculty’s medical education department, this section identified NARS competencies relevant to the training and included self-reported questions to measure trainees’ self-efficacy related to these standards. For example, participants rated their confidence in performing tasks such as “Take and record a structured, patient-centered history” under NARS Competency I: The Graduate as a Healthcare Provider; “Ensure the confidentiality and privacy of patients’ information” under NARS Competency III: The Graduate as a Professional; and “Recognize the role of other healthcare professionals in patient management” under NARS Competency V: The Graduate as a Member of the Health Team and Part of the Healthcare System. Responses were measured on a 5-point Likert scale ranging from “Not at all confident” to “Extremely confident.”

The third section focused on assessing the effect of SST24 on trainees’ clinical empathy, aligned with NARS Competency I’s emphasis on adopting an empathic and holistic approach to patient care. This section utilized the Medical Student Version of the Jefferson Scale of Empathy (JSE-S), a validated 20-item instrument designed to measure empathy among medical students [20]. Participants rated their level of agreement with each item on a 7-point Likert scale, yielding a total score ranging from 20 to 140. Higher scores indicated greater empathy levels. The JSE-S has demonstrated robust validity and reliability, with a Cronbach’s alpha coefficient of 0.80 among Jefferson Medical students [21].

The fourth section assessed participants' knowledge of core clinical topics specific to their assigned department. It included four questions for each department focusing on common diseases, investigations, and treatments. The training organizers designed it in coordination with the department teaching staff to align with the department’s intended learning outcomes and the clinical experiences encountered during the training. All questions underwent content validation by teaching faculty from each respective department to confirm their relevance to the upcoming training.

The final assessment tool was administered online through Google Forms at both the beginning and end of the training, with summed section scores used to quantify pre- to post-training changes. An English version of the assessment is provided as supplementary material (Supplementary material 2).

### Statistical analysis

Data were analyzed using Jamovi version 2.3.28. Descriptive statistics for categorical variables were presented as frequencies and percentages, while continuous variables were summarized using means and standard deviations (SD). Departments were classified into two broad categories: 'People-Oriented' and 'Technology and Procedure-Oriented,' a distinction commonly used in medical education research to ease result interpretation [22, 23]. Departments where trainees frequently interacted with patients were grouped under 'People-Oriented' department, while departments that exposed trainees to more technical and procedural aspects of medicine were categorized as 'Technology and Procedure-Oriented' specialties.

Responses to the 5-point Likert scale questions were assigned numerical values as follows: 'Extremely confident' = 5, 'Very confident' = 4, 'Moderately confident' = 3, 'Slightly confident' = 2, and 'Not at all confident' = 1, and summarized using means, SDs, and 95% confidence intervals (CIs). A paired-sample t-test was conducted to compare pre- and post-training scores. Cohen's d was calculated to assess the effect size and evaluate the practical significance of the observed differences. A *p*-value of less than 0.05 was considered statistically significant. Only trainees who completed the training’s certification requirements were included in the study’s analysis.

## Results

A total of 652 medical students participated in the SST24 program, of whom 535 met the study's inclusion criteria, yielding a response rate of 82%. Participants had a balanced gender distribution (49.2% male, 50.8% female) and an average age of 21 years (range: 18–25). The majority were third-year students (40.7%), followed by fourth-year students (31.4%) and second-year students (27.9%). Of the participants, 38.9% were assigned to People-Oriented departments (average of 29.5 training hours), while 61.1% were placed in Technology- and Procedure-Oriented departments (average of 32.8 training hours) (Table 1).

**Table 1:** SST24 trainees' characteristics and average training hours (*N* = 535)

**Characteristics**		**Training hours** **mean (SD)**
Sex *n* (%)		
Male	263 (49.2%)	30.8 (10.4)
Female	272 (50.8%)	32.2 (11.4)
Age In years mean (SD)	20.9 (0.99)	31.5 (10.9)
Academic year *n* (%)		
2nd year	149 (27.9%)	30.6 (11.3)
3rd year	218 (40.7%)	30.9 (10.7)
4th year	168 (31.4%)	33 (10.8)
Training departments *n* (%)		
People-oriented	208 (38.9%)	29.5 (11.2)
Technology-oriented	327 (61.1%)	32.8 (10.5)

*SD *Standard Deviation

Contributions to trainees’ self-efficacy in performing tasks aligned with the assessed NARS competencies are summarized in Table 2 (People-Oriented departments) and Table 3 (Technology and Procedure-Oriented departments).

**Table 2:** SST24 people-oriented departments trainees’ pre- and post-training NARS self-efficacy scores (*n* = 208)

Competencies	*n*^1^	Pre-training Mean (SD)	Post-training Mean (SD)	Mean Difference (95% CI)	*p*-value	cohen's d (95% CI)
Competency I: As a Healthcare Provider						
Take and record a structured, patient-centered history	161	3.47 (1.08)	4.25 (0.85)	0.78 (0.61, 0.96)	** <.001**	0.68 (0.51, 0.85)
Develop an empathetic approach to patients and their problems^2^	204	108 (11.5)	108 (12.9)	−0.29 (−1.73, 1.13)	0.68	−0.03 (−0.17, 0.11)
Perform appropriately-timed full physical examination of patients	124	3.06 (1.14)	3.66 (1.14)	0.61 (0.41, 0.81)	** <.001**	0.54 (0.35, 0.73)
Adopt strategies and apply measures that promote patient safety	29	2.79 (1.08)	4.1 (0.67)	1.31 (0.96, 1.66)	** <.001**	1.41 (0.89, 1.92)
Respect patients' rights, and involve them in management decision	125	4.08 (0.96)	4.3 (0.81)	0.23 (0.05, 0.40)	**0.011**	0.23 (0.05, 0.41)
Overall	125	3.49 (0.75)	4.13 (0.56)	0.65 (0.52, 0.77)	** <.001**	0.79 (0.62, 0.97)
Competency II: As a Health Promoter						
Identify the major health risks related to speciality diseases	90	2.87 (0.96)	3.8 (1.05)	0.93 (0.69, 1.18)	<.001	0.79 (0.55, 1.02)
Adopt suitable measures for Infection Control	53	3.74 (1.02)	4.26 (0.84)	0.53 (0.24, 0.82)	** <.001**	0.50 (0.22, 0.79)
Overall	90	3.14 (0.83)	3.99 (0.77)	0.85 (0.10, 1.04)	** <.001**	0.93 (0.68, 1.17)
Competency III: As a Professional						
Ensure confidentiality and privacy of patients' information	204	4.25 (0.85)	4.44 (0.74)	0.20 (0.07, 0.33)	**0.003**	0.21 (0.07, 0.35)
Adhere to professional standards and laws governing practice, and abide to the national code of ethics issued by the Egyptian Medical Syndicate	120	3.63 (1.15)	4.20 (0.99)	0.58 (0.34, 0.81)	** <.001**	0.45 (0.26, 0.64)
Exhibit appropriate professional behaviors and relationships in all aspects of practice	204	3.75 (0.91)	4.26 (0.77)	0.51 (0.07, 0.38)	** <.001**	0.54 (0.40, 0.69)
Overall	204	3.94 (0.69)	4.32 (0.62)	0.38 (0.28, 0.48)	** <.001**	0.53 (0.38, 0.67)
Competency IV: As a Scholar and Scientist						
Describe normal body structure, mechanisms maintaining homeostasis, normal development	193	3.61 (0.92)	4.13 90.82)	0.52 (0.38, 0.66)	** <.001**	0.52 (0.37, 0.67)
Describe Various causes of illness, drug actions	114	2.96 (1.12)	4.17 (0.82)	1.21 (1.01, 1.41)	** <.001**	1.11 (0.87, 1.34)
Demonstrate practical skills and procedures relevant to future practice	186	2.75 (1.08)	3.98 (0.92)	1.24 (1.08, 1.40)	** <.001**	1.11 (0.92, 1.29)
Overall	193	3.17 (0.65)	4.09 (0.55)	0.93 (0.84, 1.02)	** <.001**	1.47 (1.26, 1.67)
Competency V: As a Health team member and part of the healthcare system						
Documenting health records or electronic medical records	109	3.63 (0.94)	4.17 (0.91)	0.54 (0.32, 0.76)	** <.001**	0.47 (0.27, 0.67)
Recognize the role of other healthcare professionals in patient's management	96	3.89 (1.10)	4.40 (0.80)	0.51 (0.29, 0.73)	** <.001**	0.46 (0.25, 0.67)
Apply leadership skills to enhance team functioning and the learning environment	194	3.34 (0.99)	4.13 (0.83)	0.79 (0.65, 0.93)	** <.001**	0.81 (0.64, 0.97)
Communicate clearly, sensitively and effectively with patients	150	3.45 (0.95)	4.21 (0.79)	0.76 (0.59, 0.93)	** <.001**	0.71 (0.54, 0.90)
Overall	194	3.48 (0.7)	4.15 (0.64)	0.68 (0.58, 0.79)	** <.001**	0.93 (0.76, 1.11)

^1^Questions in each department’s assessment varied according to the clinical experiences trainees were exposed to during their training

^2^Measured using the Jefferson Scale of Empathy – Student Version (JSE-S; range: 20–140); *CI* Confidence Interval, *SD *Standard Deviation

**Table 3:** SST24 technology and procedure-oriented departments trainees’ pre- and post-training NARS self-efficacy scores (*n* = 327)

Competencies	*n*^1^	Pre-training Mean (SD)	Post-training Mean (SD)	Mean Difference (95% CI)	*p*-value	cohen's d (95% CI)
Competency I: As a Healthcare Provider						
Take and record a structured, patient-centered history	289	3.26 (1.20)	4.14 (0.89)	0.88 (0.72, 1.03)	** <.001**	0.66 (0.53, 0.79)
Develop an empathetic approach to patients and their problems^2^	344	109 (12.5)	106 (14.2)	−2.34 (−3.57, −1.11)	** <.001**	−0.20 (−0.31, −0.09)
Perform appropriately-timed full physical examination of patients	187	2.85 (1.16)	3.79 (1.05)	0.94 (0.74, 1.13)	** <.001**	0.69 (0.52, 0.84)
Adopt strategies and apply measures that promote patient safety	25	2.44 (1.33)	4.08 (1.04)	1.64 (1.07, 2.21)	** <.001**	1.19 (0.67, 1.70)
Respect patients' rights, and involve them in management decision	150	4.05 (0.98)	4.26 (0.87)	0.21 (0.03, 0.38)	**0.011**	0.19 (0.03, 0.35)
Overall	289	3.30 (0.92)	4.12 (0.72)	0.83 (0.70, 0.95)	** <.001**	0.78 (0.64, 0.91)
Competency II: As a Health Promoter						
Identify the major health risks related to speciality diseases	24	3.33 (1.13)	4.08 (1.25)	0.75 (0.12, 1.38)	** <.001**	0.51 (0.08, 0.93)
Adopt suitable measures for Infection Control	59	3.76 (1.15)	4.25 (0.94)	0.49 (0.14, 0.85)	** <.001**	0.36 (0.10, 0.62)
Overall	59	3.64 (1.05)	4.13 (0.9)	0.49 (0.16, 0.83)	** <.001**	0.38 (0.12, 0.65)
Competency III: As a Professional						
Ensure confidentiality and privacy of patients' information	327	4.08 (1.04)	4.44 (0.72)	0.36 (0.25, 0.47)	** <.001**	0.36 (0.24, 0.47)
Adhere to professional standards and laws governing practice, and abide to the national code of ethics issued by the Egyptian Medical Syndicate	245	3.56 (1.29)	4.28 (0.4)	0.72 (0.55, 0.89)	** <.001**	0.54 (0.40, 0.67)
Exhibit appropriate professional behaviors and relationships in all aspects of practice	323	3.66 (1.0)	4.15 (0.81)	0.49 (0.38, 0.59)	** <.001**	0.52 (0.40, 0.63)
Overall	327	3.79 (0.73)	4.29 (0.57)	0.51 (0.44, 0.58)	** <.001**	0.77 (0.65, 0.89)
Competency IV: As a Scholar and Scientist						
Describe normal body structure, mechanisms maintaining homeostasis, normal development	219	3.46 (1.05)	4.11 (0.84)	0.64 (0.50, 0.79)	** <.001**	0.61 (0.46, 0.75)
Describe Various causes of illness, drug actions	129	3.11 (1.15)	4.02 (0.96)	0.92 (0.71, 1.12)	** <.001**	0.79 (0.59, 0.99)
Demonstrate practical skills and procedures relevant to future practice	285	2.71 (1.09)	3.92 (0.89)	1.21 (1.07, 1.35)	** <.001**	1.01 (0.87, 1.15)
Overall	285	3.05 (0.79)	4.02 (0.59)	0.97 (0.87, 1.07)	** <.001**	1.14 (0.99, 1.29)
Competency V: As a Health team member and part of the healthcare system						
Documenting health records or electronic medical records	151	3.48 (1.06)	4.17 (0.86)	0.70 (0.50, 0.80)	** <.001**	0.56 (0.39, 0.73)
Recognize the role of other healthcare professionals in patient's management	31	4.16 (0.94)	4.55 (0.77)	0.39 (0.00, 0.77)	**0.05**	0.37 (0.00, 0.73)
Apply leadership skills to enhance team functioning and the learning environment	295	3.41 (1.08)	4.06 (0.87)	0.64 (0.53, 0.76)	** <.001**	0.62 (0.50, 0.75)
Communicate clearly, sensitively and effectively with patients	236	3.49 (1.07)	4.14 (0.92)	0.64 (0.49, 0.80)	** <.001**	0.53 (0.39, 0.66)
Overall	295	3.48 (0.77)	4.13 (0.64)	0.64 (0.55, 0.74)	** <.001**	0.81 (0.68, 0.94)

^1^Questions in each department’s assessment varied according to the clinical experiences trainees were exposed to during their training

^2^Measured using the Jefferson Scale of Empathy – Student Version (JSE-S; range: 20–140); *CI* Confidence Interval, *SD *Standard Deviation

### Competency I: 'The graduate as a healthcare provider'

A significant overall improvement in self-efficacy was reported by both People-Oriented departments trainees (Cohen's d = 0.79, CI: 0.62–0.97) and Technology and Procedure-Oriented departments trainees (Cohen's d = 0.78, CI: 0.64–0.91). The most notable improvements were seen in trainees' confidence in taking patient histories, conducting physical examinations, and promoting patient safety (all *p*-values < 0.001). However, a slight decline in self-reported empathy toward patients was noted among trainees from Technology and Procedure-Oriented departments (Cohen's d = −0.2, CI: −0.31 to −0.09).

### Competency II: 'The graduate as a health promoter'

Trainees in both People-Oriented and Technology and Procedure-Oriented departments reported significant increases in their confidence in identifying health risks and adopting infection control measures (*p*-values < 0.001). However, trainees in People-Oriented departments demonstrated a greater self-efficacy increase (Cohen's d = 0.93, CI: 0.69–1.17) compared to those in Technology and Procedure-Oriented departments (Cohen's d = 0.38, CI: 0.12–0.65).

### Competency III: 'The graduate as a professional'

Trainees in Technology and Procedure-Oriented departments showed a greater increase in self-efficacy related to professionalism (Cohen's d = 0.77, CI: 0.65–0.89) compared to those in People-Oriented departments (Cohen's d = 0.53, CI: 0.38–0.67), particularly in their confidence in ensuring confidentiality and patient privacy (MD: 0.36, CI: 0.25–0.47 vs. 0.20, CI: 0.07–0.33, respectively).

### Competency IV: 'The graduate as a scholar and scientist'

Trainees in both People-Oriented and Technology and Procedure-Oriented departments reported significant increases in their confidence in describing normal body structure, various illnesses, and demonstrating practical skills (all *p*-values < 0.001). Specifically, trainees in Technology and Procedure-Oriented departments reported greater confidence in describing human anatomy (Cohen's d = 0.61, CI: 0.46–0.75 vs. 0.52, CI: 0.37–0.67),while People-Oriented department trainees reported a more pronounced increased confidence in describing the causes of illnesses and drug actions (Cohen’s d = 1.11, CI: 0.87–1.34 vs. 0.79, CI: 0.59–0.99).

### Competency V: 'The graduate as a health team member'

Significant self-efficacy increases were observed in both People-Oriented and Technology and Procedure-Oriented departments, particularly in trainees' self-efficacy in communicating clearly with patients (MD: 0.76, CI: 0.59–0.93 for People-Oriented; MD: 0.64, CI: 0.49–0.80 for Technology and Procedure-Oriented, both *p* < 0.001) and applying leadership skills to enhance team functioning and the learning environment (MD: 0.79, CI: 0.65–0.93 for People-Oriented; MD: 0.64, CI: 0.53–0.76 for Technology and Procedure-Oriented, both *p* < 0.001).

### Clinical knowledge acquisition

**Fig. 2:**
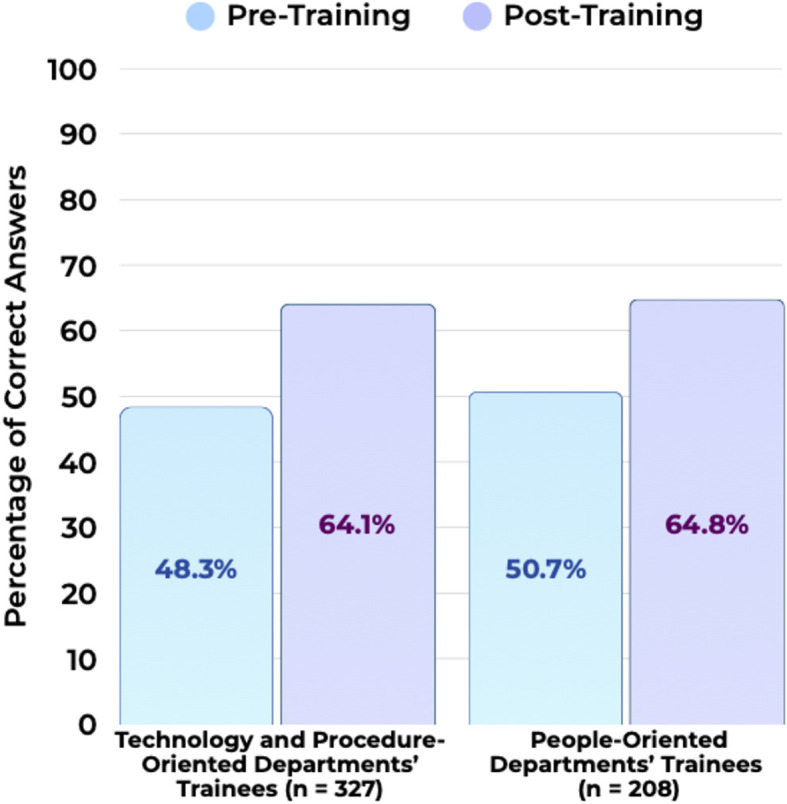
Pre- and post-training percentage of correct answers to clinical knowledge questions for SST24 trainees in technology- and people-oriented departments

Participants in both department types demonstrated improvement in their clinical knowledge scores from pre- to post-training. In Technology and Procedure-Oriented departments, the mean correct answers increased from 48.3% pre-training to 64.1% post-training. Similarly, in People-Oriented departments (*n* = 207), the mean correct answers improved from 50.7% to 64.8% (Fig. 2).

## Discussion

Healthcare providers are fundamental to the well-being of any nation, a reality particularly pronounced in LMICs. Consequently, medical schools have embraced CBME and established rigorous standards for their graduates. However, ensuring that these graduates are adequately equipped to meet these standards presents a formidable challenge, particularly in contexts constrained by limited resources. This underscores the pressing need for initiatives that supplement medical education, ensuring the development of competent and capable healthcare providers. This study aimed to assess the effectiveness of one such initiative, the student-led SST24 program, in enhancing trainees’ self-efficacy and contributing to the NARS.

A key feature of the SST program is its structure as a student-led initiative rooted in the principles of CBME. This model is part of a global movement in health professions education, with various successful implementations in both high-resource and LMIC settings. For example, a recent evaluation of a student-led clinical learning environment in the UK found that the model enhanced students' confidence, knowledge, and professional development [9]. Similarly, a study of a longstanding student-run free clinic in South also suggests that such initiatives are valuable for improving students' clinical skills while providing essential community care [8]. These initiatives not only foster innovation but also equip students with essential soft skills, as well as leadership and managerial competencies, crucial for their future roles in healthcare. For instance, Scott and Swartz found that serving on the boards of student-led clinics significantly enhanced students' leadership skills [24]. Likewise, Black et al. [25], Bostick et al. [26], and Kavanagh et al. [27] reported increased professional pride among students participating in such clinics. Schutte et al. further emphasized that competencies are best developed in settings mirroring professional practice, with student-led clinics fostering skills in responsibility, patient care, and interprofessional collaboration [28].

SST contributed notably to trainees’ self-efficacy across multiple NARS competency domains. Participants reported significant improvements in their self-efficacy for patient communication (Competency I: The Graduate as a Healthcare Provider), professionalism and confidentiality (Competency III: The Graduate as a Professional), and teamwork skills (Competency V: The Graduate as a Member of the Health Team and Part of the Healthcare System). They also reported increased self-efficacy in performing clinical tasks, such as history taking and physical examinations, further reinforcing their development in Competency I. These findings align with a 4-week primary health care center training program at Arabian Gulf University [29], which reported substantial improvements in students’ self-efficacy in clinical skills compared to their baseline abilities. Similarly, our results mirror those of a 12-week preparation for internship course in New South Wales [30] and that of a one-day multidisciplinary workshop in Romania [31], where students reported enhanced confidence and perceptions of their capabilities in procedural skills, operational management, and administrative tasks.

The enhanced clinical competence observed in both the SST program and other hospital-situated medical student training programs can be explained through the concept of 'supported participation in practice.' In a synthesis of 168 empirical studies, Dornan et al. concluded that clinical learning is a dynamic interaction between students, patients, and doctors [32]. This concept is further supported by a study that similarly identified supported participation in practice as the core aspect of medical students' clinical learning [33]. When provided with adequate support from practitioners, students transition from passive observation to active performance, gradually acquiring the practical competencies and confidence needed for effective practice [34]. It is through this model that, in the context of medical education with reduced opportunities for participation in clinical practice due to an increase in the number of medical students, SST plays an essential role in contributing to the development of competent healthcare providers.

The decline in trainees' self-rated empathy, a key component of the affective domain of learning, warrants careful consideration. While our program aimed to enhance cognitive and psychomotor skills, this finding suggests that certain learning environments can negatively impact affective development [35]. This phenomenon is not uncommon in medical education research. Multiple studies have observed a reduction in empathy following clinical exposure, particularly in departments where the emphasis on technical skills (the psychomotor domain) may overshadow the development of long-term patient-physician relationships (the affective domain) [36]. For instance, a year-long longitudinal study on medical students involved in service-learning found a decline in empathy, especially in terms of understanding patients' perspectives and providing compassionate care [37]. In contrast, a longitudinal study assessing the impact of a Longitudinal Integrated Clerkship (LIC) showed that participation in the LIC helped buffer the decline in empathy, unlike the experiences of students in more traditional clerkship models [38].

The decrease in trainees' self-rated empathy after the training may be attributed to the nature of the teaching hospitals where the training took place. In these hospitals, the patient load far exceeds the number of healthcare providers, leading to a strained healthcare system where time and resources for meaningful patient interactions are limited. This issue is reflected in a study conducted among residents in Egypt, where 83% reported an inappropriate patient-staff ratio, and 85% expressed dissatisfaction with their relationships with patients [39]. The overwhelming workload creates a high-pressure environment which can limit opportunities for empathetic engagement with patients [40]. Furthermore, poor role modeling, another factor known to impact empathy development, may have resulted from staff being overburdened rather than lacking empathy in patient interactions [41]. In light of these challenges, future iterations of the training could consider integrating workshops that help reinforce the importance of empathy in patient interactions, which have been shown to effectively increase empathy in medical students [42].

Improvements in department-specific knowledge related to diseases, investigations, and treatments highlight the program’s ability to align trainees' learning experiences with clinical exposure. By immersing students in real clinical environments and providing adequate support from departmental teaching staff, the program fosters active reflection and the application of theoretical knowledge to practice. These findings are consistent with a study at McGill [43], where an integrated week in geriatric medicine was found to be more effective than 10 weekly sessions as an introductory experience for medical students. They also align with the results of a one-week early clinical exposure program for pre-medical students in India [44], which demonstrated how experiential learning encouraged reflection on applying basic sciences to clinical practice, enhancing knowledge across various clinical domains. Overall, these results underscore the program's role in bridging the gap between theoretical learning and clinical practice, better preparing students for their future roles in healthcare.

The inclusion of supplementary workshops enriched the training experience by addressing critical aspects of medical practice often overlooked in traditional curricula. Topics such as history-taking, managing emergency situations, and the ethical aspects of medicine contributed directly to NARS competencies while equipping trainees with essential interpersonal skills for professional development. In parallel, Sutoi et al. reported that their one-day workshop significantly elevated students’ practical knowledge and self-assessed leadership and teamwork abilities, regardless of their year of training [31]. They employed a similar hands-on, team-based learning model with large effect sizes, confirming that short, focused interventions can effectively enhance both technical and non-technical competencies. Additionally, our integration of reflective portfolios expanded on these workshops, encouraging trainees to critically evaluate their experiences, foster self-awareness, and apply lessons learned to future clinical scenarios. Reflective practice, as demonstrated in other studies [45, 46], not only enhances clinical and communication skills but also promotes a deeper understanding of healthcare providers' roles and responsibilities.

SST’s potential extends beyond its local implementation. By relying on student organizers, peer-led supervision, and existing faculty infrastructure, SST minimizes faculty instructional hours and associated institutional costs, offering a relatively cost-efficient alternative to traditional, faculty-intensive clinical training models in resource-limited settings. Importantly, many student supervisors were once trainees themselves, and several teaching staff members previously served as SST supervisors or participated in earlier rounds of the training. This cyclical structure fosters continuity and allows new team members to build on the experiences of previous cohorts, while also maintaining familiarity with the program among both students and staff. After being held yearly for over a decade, the program has evolved through continuous annual improvements, demonstrating both sustainability and adaptability. As one of the many activities held within the International Federation of Medical Students Associations (IFMSA), SST also serves as a blueprint that can inspire similar student-driven initiatives globally, especially in low- and middle-income countries aiming to strengthen competency-based medical education despite infrastructural limitations.

### Strengths and limitations

SST’s key strengths include its student-led, relatively cost-efficient structure, which fosters leadership and organizational skills among the organizing team while promoting growth in trainees’ self-efficacy. Furthermore, by placing students at the center of program design and delivery, it aligns with emerging accreditation standards that emphasize youth participation in education, reduces the resource burden on medical institutions, and empowers students as active partners in their own learning.

However, a primary limitation in the program, and thus the study, is its reliance on self-reported data to measure outcomes. While participants reported significant gains in self-efficacy, self-perception may not correlate with actual performance, potentially leading to an overestimation of the program's true effectiveness. A further limitation is the lack of formal psychometric validation for our self-developed assessment questionnaire. The observational design without a control group also limits causal inference, and the study's focus on a single institution and specific year groups reduces generalizability. Future evaluations and adaptations of SST should incorporate objective, externally-assessed measures, such as tutor-led Objective Structured Clinical Examinations (OSCEs), to validate these self-reported self-efficacy gains. Finally, while SST offers a resource-efficient model in our context, primarily due to voluntary faculty time and a student-led structure, we acknowledge that what is considered 'low-cost' is context-dependent and may not translate to all healthcare education systems where such supervision is highly resourced.

## Conclusion

The findings of this study suggest that a student-led program like SST24 is associated with significant gains in trainees' self-efficacy toward NARS competencies across multiple domains. Trainees reported significant improvements in their self-reported confidence to communicate effectively, maintain patient confidentiality, and apply professionalism. However, there was a slight decrease in empathy, highlighting the complexity of developing affective traits like empathy in medical education.

SST exemplifies how student-led programs can complement formal medical education by providing opportunities for clinical exposure, peer learning, and reflective practice. By adapting the program’s structure and activities to different institutional contexts, SST could serve as a model for addressing similar challenges in clinical training worldwide, particularly in resource-constrained settings.

## Supplementary Information

Supplementary Material 1.

Supplementary Material 2.

## Data Availability

The data of this study is available from the corresponding author upon reasonable request.

## Abbreviations

CBME: Competency-based medical education
CI: Confidence interval
ILO: Intended learning outcome
IRB: Institutional research board
IFMSA: International Federation of Medical Students Associations
LIC: Longitudinal Integrated Clerkship
LMIC: Low- and middle-income countries
MD: Mean difference
MSSA: Mansoura Students’ Scientific Association
NARS: National Academic Reference Standards
OSCE: Objective Structured Clinical Examination
SST: Shouman Summer Training
