# Supplementary material for: Enhancing medical students’ self-efficacy toward national competency standards: a student-led training model in a lower-middle-income country
Source: BMC Med Educ. 2025 Dec 8;25:1687. doi: 10.1186/s12909-025-08335-9 (PMC12690811; doi:10.1186/s12909-025-08335-9)
Supplement: Supplementary file 1 — Supplementary Material 1. [file 12909_2025_8335_MOESM1_ESM.docx]

**List of departments, classification, trainee distribution, and average training hours.**

| **Department** | **Classification** | **1st Wave  Trainees (n)** | **1st wave Supervisors (n)** | **Training hours  mean (SD)** | **2nd Wave  Trainees (n)** | **2nd wave supervisors (n)** | **Training hours  mean (SD)** |
| --- | --- | --- | --- | --- | --- | --- | --- |
| Anesthesia and surgical ICU | Technology and Procedure-Oriented | 6 | 1 | 47.7 (2.06) | 6 | 1 | 34.4 (3.3) |
| Cardio-thoracic Surgery | Technology and Procedure-Oriented | 12 | 2 | 28.5 (2.03) | 12 | 2 | 30.21 (1.96) |
| Cardiology | People-Oriented | 12 | 1 | 22.6 (0.93) | 10 | 1 | 17.5 (1.8) |
| Chest | People-Oriented | 8 | 1 | 51.9 (3.76) | 8 | 1 | 26.5 (3.25) |
| Dermatology and Andrology | People-Oriented | 8 | 1 | 18.8 (2.02) | 8 | 1 | 20.8 (0.95) |
| Emergency hospital | Technology and Procedure-Oriented | 16 | 2 | 44.9 (2.93) | 16 | 2 | 23.23 (1.92) |
| Endocrinology | People-Oriented | 8 | 1 | 19.3 (1.5) | 8 | 1 | 20.93 (3.09) |
| ENT | Technology and Procedure-Oriented | 15 | 2 | 22 (2.34) | 15 | 2 | 26.25 (2.77) |
| Gastroenterology and Hepatology | People-Oriented | 8 | 1 | 23.57 (2.07) | 8 | 1 | 28.17 (2.14) |
| Gastrointestinal Surgery | Technology and Procedure-Oriented | 12 | 2 | 29.5 (3.84) | 12 | 2 | 35.45 (5.68) |
| General Surgery | Technology and Procedure-Oriented | 9 | 1 | 43 (6.3) | 9 | 1 | 38.29 (2.14) |
| Geriatric medicine | People-Oriented | 6 | 1 | 28 (3.08) | 6 | 1 | 21.42 (0.92) |
| Gynecology center | People-Oriented | 12 | 2 | 48.5 (2.42) | 12 | 2 | 45.9 (3.51) |
| Hematology | Technology and Procedure-Oriented | 6 | 1 | 19.1 (2.92) | 6 | 1 | 15.7 (1.99) |
| Medical ICU | Technology and Procedure-Oriented | 8 | 1 | 30.12 (2.58) | 8 | 1 | 32 (3.01) |
| Medical oncology | People-Oriented | 8 | 1 | 36.07 (3.25) | 8 | 1 | 40.3 (2.43) |
| Nephrology | People-Oriented | 6 | 1 | 19.67 (2.11) | 6 | 1 | 24.2 (1.64) |
| Neurology | People-Oriented | 8 | 1 | 20 (0.3) | 8 | 1 | 29.9 (6.4) |
| Neurosurgery | Technology and Procedure-Oriented | 8 | 1 | 34.4 (1.82) | 8 | 1 | 43.67 (2.66) |
| Oncology | Technology and Procedure-Oriented | 18 | 2 | 40.7 (5.67) | 18 | 2 | 32.3 (4.07) |
| Ophthalmology | Technology and Procedure-Oriented | 14 | 2 | 27.3 (1.48) | 14 | 2 | 28.44 (3.84) |
| Orthopedic Surgery | Technology and Procedure-Oriented | 15 | 2 | 36.64 (6.09) | 15 | 2 | 35.6 (1.26) |
| Pediatric Medicine | People-Oriented | 12 | 2 | 46 (3.94) | 12 | 2 | 26 (2.92) |
| Pediatric surgery | Technology and Procedure-Oriented | 12 | 2 | 21.54 (1.78) | 12 | 2 | 21.32 (2.18) |
| Plastic Surgery | Technology and Procedure-Oriented | 18 | 2 | 39.03 (4.7) | 18 | 2 | 38.57 (4.13) |
| Psychiatry | People-Oriented | 14 | 2 | 15 (1.05) | 14 | 2 | 25.32 (3.27) |
| Radiology | Technology and Procedure-Oriented | 8 | 1 | 50 (4.47) | 8 | 1 | 32 (2.58) |
| Rheumatology and Rehabilitation | People-Oriented | 8 | 1 | 32.9 (3.13) | 8 | 1 | 32.5 (2.26) |
| Toxicology | Technology and Procedure-Oriented | 8 | 1 | 32.5 (2.95) | 8 | 1 | 10.5 (1.2) |
| Urology | Technology and Procedure-Oriented | 16 | 4 | 43.37 (6.9) | 16 | 4 | 40.14 (15.59) |
| Vascular Surgery | Technology and Procedure-Oriented | 8 | 2 | 12.2 (9.52) | 8 | 2 | 21.14 (2.54) |
| **Total** |  | **327** | **47** | **-** | **325** | **47** | - |
