# Supplementary material for: Enhancing medical students’ self-efficacy toward national competency standards: a student-led training model in a lower-middle-income country
Source: BMC Med Educ. 2025 Dec 8;25:1687. doi: 10.1186/s12909-025-08335-9 (PMC12690811; doi:10.1186/s12909-025-08335-9)
Supplement: Supplementary file 2 — Supplementary Material 2. [file 12909_2025_8335_MOESM2_ESM.docx]

**Section 1- Personal information**

Age:

Sex:

Educational year for the last term 2023/2024?

Assigned department:

**Section 2- NARS competencies**

**Please indicate your level of confidence in applying each of the following by selecting a number from 1 to 5, where:** 1 – Not at all confident, 2 – Slightly confident, 3 – Moderately confident, 4 – Very confident, 5 – Extremely confident.

- Take and record a structured, patient-centered history
- Develop an empathetic approach to patients and their problems
- Perform appropriately-timed full physical examination of patients
- Adopt strategies and apply measures that promote patient safety
- Respect patients' rights, and involve them in management decision
- Identify the major health risks related to speciality diseases (specific per-department)
- Adopt suitable measures for Infection Control
- Ensure confidentiality and privacy of patients' information
- Adhere to professional standards and laws governing practice, and abide to the national code of ethics issued by the Egyptian Medical Syndicate
- Exhibit appropriate professional behaviors and relationships in all aspects of practice
- Describe normal body structure, mechanisms maintaining homeostasis, normal development
- Describe Various causes of illness, drug actions (specific per-department)
- Demonstrate practical skills and procedures relevant to future practice (specific per-department, i.e: Measuring blood pressure and vital signs, reading and interpreting ECGs, handling medical instruments)
- Documenting health records or electronic medical records
- Recognize the role of other healthcare professionals in patient's management
- Apply leadership skills to enhance team functioning and the learning environment
- Communicate clearly, sensitively and effectively with patients

**Section 3- Jefferson Scale of Empathy Medical Student Version** (available from: https://www.jefferson.edu/academics/colleges-schools-institutes/skmc/research/research-medical-education/jefferson-scale-of-empathy.html)

**Section 4: Department-specific clinical knowledge questions:**

**Cardiology department
The first line of TTT for patient MI (providing we are in MUH):**○ Giving anticoagulants
○ Giving thrombolytics
○ Catheterization
○ I don't know

**Which of the following is considered as a type of Heart failure:**○ Arrhythmic HF
○ Diastolic HF
○ "HIS" heart failure
○ I don't know

**Which of the following is considered as the power of all the heart problems:**○ LDL
○ Steroids
○ Alcohols
○ I don't know

**"WilliaM MarroW" is a mnemonic used for remembering the:**○ RBBB & LBBB
○ ECG intervals
○ Blood dynamics
○ I don't know

**Dermatology and Andrology department
In chronic eczema skin lines are:**○ Exaggerated
○ Undefined
○ Defined
○ I don't know

**What happens to hair in androgenic alopecia:**○ Falling
○ Thinning
○ Breaking of hair follicle
○ I don't know

**Patient with milky white patch treated by:**○ Excimer
○ Cryotherapy
○ Topical steroid
○ I don't know

**Treatment of aquagenic pruritis:**○ Antihistamine
○ Antibiotic
○ Antifungal
○ I don't know

**Endocrinology department
In diabetic patients the most common infection is:**○ Staph aureus
○ Tenia pedis
○ Varicella
○ I don't know

**Drug used to treat diabetic neuropathy:**○ Gapentine
○ Thaiotacid
○ Gapentine and Thaiotacid
○ I don't know

**Most common drug used in type 2 DM:**○ Metformin
○ Canaglifazone
○ Gliptins
○ I don't know

**Gold standard investigation for follow-up in thyroid diseases:**○ TSH
○ ACTH
○ GH
○ I don't know

**Gastroenterology and Hepatology department
Stage of chronic hepatitis means:**○ Assessment of fibrosis in the liver biopsy
○ Assessment of fatty deposition in the liver biopsy
○ Assessment of portal inflammation in the liver biopsy
○ I don't know

**Female patient 22 years old presents with abdominal pain, bloody diarrhea with bilateral knee and ankle pains. By colonoscopy, there are ulcers in the rectum and sigmoid. The diagnosis of this case is:**○ Crohn's disease
○ Ulcerative colitis
○ Intestinal obstruction
○ I don't know

**Male patient 55 years old with a history of chronic HCV for 15 years was admitted to the hospital for radiological assessment after detection of elevated alpha-fetoprotein level. What is the most probable diagnosis:**○ Alcoholic cirrhosis
○ Liver cell adenoma
○ Hepatocellular carcinoma
○ I don't know

**A 45-year-old man with a duodenal ulcer treated with a combination of drugs intended to heal the mucosal damage and to eradicate H. pylori. An antibacterial drug that is used commonly to eradicate intestinal H. pylori is:**○ Clarithromycin and amoxicillin only
○ Clarithromycin, amoxicillin, and PPI
○ Ciprofloxacin, amoxicillin, PPI
○ I don't know

**Neurology department
Hemorrhagic stroke is most likely caused by:**○ Emboli
○ HTN
○ Atherosclerosis
○ I don't know

**Parkinson’s can be treated by:**○ L-dopa
○ Carbamazepine
○ Valproate
○ I don't know

**Guillain-Barré patient is treated with:**○ Cortisol
○ Plasmapheresis
○ Monoclonal antibody
○ I don't know

**First line of treatment for a disc patient is:**○ Surgical interventions
○ Physiotherapy
○ Pain control analgesics
○ I don't know

**Pediatric Medicine department
Which of the following is the most common cause of bacterial pneumonia in children?**○ Streptococcus pneumoniae
○ Mycoplasma pneumoniae
○ Haemophilus influenzae
○ Staphylococcus aureus
○ I don't know

**At what age is the first dose of the measles, mumps, and rubella (MMR) vaccine typically administered?**○ At birth
○ 2 months
○ 6 months
○ 12-15 months
○ I don't know

**What is the normal range for the respiratory rate in a newborn?**○ 20-30 breaths per minute
○ 30-60 breaths per minute
○ 40-70 breaths per minute
○ 50-80 breaths per minute
○ I don't know

**Which of the following is a common symptom of acute otitis media in children?**○ Abdominal pain
○ Ear pain
○ Joint pain
○ Sore throat
○ I don't know

**A 2-day-old baby who is at home and looks visibly jaundiced should:**○ Be admitted to the hospital for exclusion of sepsis
○ Have phototherapy in hospital
○ Be placed in the sunlight, which converts unconjugated bilirubin to a safer form
○ Be placed under phototherapy, which converts unconjugated to conjugated bilirubin
○ Should have their bilirubin measured
○ I don't know

**Gynecology Center department
Drug (............) increases fetal lung surfactant and decreases the possibility of (.........):**○ Aspirin – fetal blood disorders
○ Garamycin – fetal rashing
○ Dexamethazone – Respiratory distress syndrome
○ I don’t know

**In nullipara females, dilatation occurs at the rate of approximately …….cm/h:**○ 4
○ 12
○ 1
○ I don't know

**Fetal viability can be detected at……week by US:**○ 7th
○ 20th
○ 12th
○ I don't know

**We differentiate preeclampsia from gestational HTN by the presence of ……in urine:**○ CHO
○ Protein
○ Pus cells
○ I don’t know

**Recommended procedure for ectopic pregnancy past 20 weeks:**○ Vaginal delivery
○ Cesarean section
○ Termination
○ I don’t know

**Nephrology department
The only time we use creatinine levels as an indication for dialysis is when:**○ Fever occurs
○ Its rate of increase gets too high too fast, not when the creatinine is just high
○ I don't know

**Early stage kidney disease - The mechanism of biological therapy is:**○ Stimulate immune system
○ Cytokines suppression
○ Inhibition of neutrophil's function
○ I don't know

**Rheumatoid always spares …… Joint:**○ Metacarpophalangeal joint (MCP)
○ Distal interphalangeal joints (DIP)
○ Proximal interphalangeal joints (PIP)
○ I don't know

**3 regions which become ulcerative due to Behcet's disease:**○ Eyes, foot, scalp
○ Mouth, eyes, foot
○ Mouth, genitalia, eyes
○ I don't know

**Chest department
Fiberoptic bronchoscopy is indicated in all the following except:**○ Massive hemoptysis
○ Interstitial lung diseases
○ Delayed resolving pneumonia
○ Lung collapse
○ I don't know

**All of the following are obstructive ventilatory defects in spirometry except:**○ Bronchial asthma
○ COPD
○ Emphysema
○ Interstitial pulmonary fibrosis
○ I don't know

**The most safe anticoagulant for pulmonary embolism in pregnant females is:**○ Warfarin
○ LMWH
○ Coumadin
○ I don't know

**Most common cause of bacterial pneumonia:**○ Pseudomonas
○ Streptococcus pneumonia
○ Klebsiella
○ Staphylococcus aureus pneumonia
○ I don't know

**Medical Oncology department
Nuclear medicine has:**○ Both
○ Therapeutic role
○ Diagnostic role
○ I don't know

**Breast cancer management depends on:**○ Pathology only
○ Staging only
○ Age of the patient only
○ Staging, pathology, immunohistochemistry, and age of the patient
○ I don't know

**Investigation for cancer cervix include:**○ CT abdomen and pelvis only
○ Biopsy only
○ MRI abdomen and pelvis only
○ MRI abdomen and pelvis, CT chest, cystoscopy, proctoscopy, and biopsy
○ I don't know

**Rheumatology and Rehabilitation department
Rheumatoid arthritis (RA) spares which of the following?:**○ DIP
○ PIP
○ MCP
○ I don't know

**Gout is a disease due to:**○ Increased glutamic acid
○ Decreased uric acid
○ Increased uric acid
○ I don't know

**Hallmark of diagnosis of Ankylosing spondylitis is:**○ Knee pain
○ Sacroiliitis
○ Uveitis
○ I don't know

**All of the following are deformities seen in RA patients except:**○ Boutonniere
○ Radial deviation of the wrist
○ Heberden nodes
○ I don't know

**1st TTT for RA is:**○ Biological treatment
○ NSAIDs
○ Methotrexate
○ I don't know

**Geriatric Medicine Department
Which of the following statements regarding Dementia is not true?**○ Dementia is a normal part of ageing, and it is not possible for persons with dementia to continue to engage and contribute within society and have a good quality of life.
○ Dementia is overwhelming for the caregivers, and adequate support is required for them from the health, social, financial, and legal systems.
○ Countries must include dementia on their public health agendas. Sustained action and coordination are required at international, national, regional, and local levels.
○ People with dementia and their caregivers often have unique insights into their condition and life.
○ People with dementia and their caregivers should be involved in formulating the policies, plans, laws, and services that relate to them.
○ I don't know

**Which of the following statements about the CGA is not true?**○ A geriatrician can complete a CGA independently.
○ CGA includes physical and mental health, environment, and social function.
○ CGA is associated with lower medication use.
○ CGA results in improved independence.
○ The goal of CGA is to maximize independence and prevent future disability.
○ I don't know

**Which of the following statements regarding CGA is not true? Elderly people admitted to hospital receiving a CGA are:**○ Less likely to be admitted to an institution.
○ Less likely to die during the acute admission.
○ Less likely to experience functional decline.
○ More likely to show benefit on cognitive measures.
○ No different from the control group at 12 months’ follow-up.
○ I don't know

**76-year-old woman was referred to the falls assessment clinic after having six falls in as many weeks. Her timed get up and go test score was 25 sec. Which of the following statements not describes the timed get up and go test?**○ It cannot be completed by people with cognitive impairment.
○ It is an unhelpful test in people with painful arthritis.
○ It is only useful in assessing the outcome of falls interventions.
○ It may be helpful in diagnosing the cause of falls.
○ The patient should be tested without using any walking aid.
○ I don't know

**Psychiatry department
Which of the following neurotransmitters is associated with feelings of pleasure and reward, as well as motivation?**○ Dopamine
○ Serotonin
○ Norepinephrine
○ I don't know

**A person experiencing intense fear or discomfort in situations where escape might be difficult is likely suffering from:**○ Panic disorder
○ Schizophrenia
○ Obsessive-compulsive disorder
○ I don't know

**Which disorder is characterized by periods of extreme excitement and energy (mania) followed by periods of depression?**○ Borderline personality disorder
○ Bipolar disorder
○ Major depressive disorder
○ I don't know

**Delusions and hallucinations are most commonly associated with which mental disorder?**○ Generalized anxiety disorder
○ Bipolar disorder
○ Schizophrenia
○ I don't know

**A mental health disorder that involves chronic and excessive worry about a variety of events and situations is known as:**○ Narcissistic personality disorder
○ Dissociative identity disorder
○ Generalized anxiety disorder
○ I don't know

**Cardio-thoracic Surgery department**

**The artery used in CABG surgery directly, not a graft, is:**○ Subclavian artery
○ Left internal mammary artery
○ Axillary artery
○ I don't know

**Cardiopulmonary bypass device is connected to:**○ Lung, SVC
○ Aorta, Right side of the heart
○ Pulmonary artery, Subclavian vein
○ I don't know

**Cardioplegia during open heart surgery was done by:**○ Hypercalcemia
○ Hyperkalemia
○ Hypocalcemia
○ I don't know

**Chest tube is inserted between:**○ 7th, 8th rib
○ 3rd, 4th rib
○ 4th, 5th rib
○ I don't know

**Medical ICU department**

**1st step in managing septic shock:**○ Resuscitation with fluid
○ Giving antibiotics
○ Giving antipyretic
○ I don't know

**ECHO investigation is indicated in which shock:**○ Hypovolemic
○ Neurogenic
○ Cardiogenic
○ I don't know

**1st line in treating bradycardia:**○ Adrenaline
○ Dopamine
○ Atropine
○ I don't know

**In hypovolemic shock, role of ultrasound is to:**○ Check the vitality of internal organs
○ See if IVC is collapsed
○ Assess kidney and urinary bladder
○ I don't know

**ENT department**

**The following is true about the tympanic membrane EXCEPT:**○ The light reflex is due to the concave position of the membrane.
○ It is placed obliquely forming acute angle with meatus anteriorly and obtuse one posteriorly.
○ It is rounded in shape.
○ I don't know

**The normal tympanic membrane is pearly white in color.
The following is true about the Eustachian tube except:**○ It ends 1 cm behind the posterior end of the inferior turbinate.
○ The upper 1/3 is bony while the lower 2/3 is fibrocartilagenous.
○ It is normally opened at rest.
○ I don't know

**Stapedectomy is one line for treatment of:**○ I don't know
○ Secretory otitis media
○ Otitic barotrauma
○ Otomycosis
○ Otosclerosis

**The ostium of the maxillary sinus opens in:**○ Between the medial wall & floor of the orbit.
○ I don't know
○ Floor of the sinus.
○ Roof of the sinus.
○ Medial wall of the sinus.

**Which is true about laryngeal carcinoma:**○ All of the above.
○ Commoner in males.
○ Is predisposed by smoking.
○ I don't know
○ The commonest type is squamous cell carcinoma.

**Gastrointestinal Surgery department**

**The radical treatment for malignant obstructive jaundice includes:**○ ERCP and stent
○ PTD
○ Double bypass
○ Whipple operation
○ Hepaticojejunostomy
○ I don't know

**The following is a side view endoscopy:**○ Esophagoscope
○ Proctoscope
○ ERCP
○ Colonoscopy
○ Gastroscopy
○ I don't know

**One of the following is a red flag in patients with GERD symptoms:**○ Heartburn
○ Dyspepsia
○ Dysphagia
○ Retrosternal pain
○ Easy fatigability
○ I don't know

**The gold standard treatment for a patient with lower rectal cancer is:**○ Neoadjuvant therapy then surgery
○ Surgery
○ Adjuvant therapy
○ Neoadjuvant therapy and watchful observation
○ Endoscopic resection
○ I don't know

**General Surgery department**

**Patients with both palmar and axillary hyperhidrosis can be treated by thoracoscopic excision of the ...... sympathetic ganglia:**○ T3 and T4
○ T1 and T2
○ T2 and T3
○ I don't know

**The subclinical hyperthyroidism is characterized by:**○ I don't know
○ Increased TSH and decreased free T3 & free T4 levels in the blood
○ Decreased TSH and increased free T3 and free T4 levels in the blood
○ Decreased TSH and normal free T3 & free T4 levels in the blood

**In varicocelectomy, which of the following is the safe approach?**○ Low approach
○ I don't know
○ High approach

**............ is one of the most common breast lumps found in young women, typically between the ages of 15 and 35:**○ Hemangioma
○ Fibroadenoma
○ Neurofibroma
○ I don't know

**Orthopedic Surgery department**

**What’s the steps of cleaning a wound?**○ I don't know
○ Saline, saline, betadine
○ Betadine, saline, saline
○ Saline, betadine, saline

**What’s the substance used to sterilize?**○ Alcohol only
○ Betadine only
○ Alcohol, betadine
○ I don't know

**Interlocking nail is used for:**○ Femur shaft fracture
○ Hand fracture
○ Shoulder dislocation
○ I don't know

**What’s the best site for spinal anesthesia?**○ L3-4
○ T6-7
○ L1-2
○ I don't know

**Surgical Oncology department**

**Which of the following is considered a locoregional treatment for cancer:**○ Chemotherapy
○ Gene therapy
○ Target therapy
○ Immunotherapy
○ I don't know
○ Radiotherapy

**Which of the following is the commonest type of Thyroid cancer:**○ Follicular
○ Lymphoma
○ I don't know
○ Anaplastic
○ Medullary
○ Papillary

**Which of the following investigations is the best for breast cancer screening:**○ Ultrasound
○ I don't know
○ Mammography
○ MRI
○ CT
○ X-ray

**All of the following are synthetic threads except:**○ Silk
○ Vicryl
○ Prolene
○ PDS
○ I don't know
○ Monocryl

**Which of the following muscles divide the Axillary lymph nodes into 3 levels:**○ Latissimus dorsi
○ Pectoralis major
○ Teres minor
○ Serratus anterior
○ I don't know
○ Pectoralis minor

**Pediatric Surgery department**

**Vomiting resulting from infantile hypertrophic pyloric stenosis is characterized by:**○ Vomiting is dating since birth
○ Non-bilious vomiting
○ Non-projectile vomiting
○ Usually not resulting in dehydration nor affecting the body weight of the infant.
○ Not related to feeds
○ I don't know

**Clinical presentations of neonatal intestinal obstruction include:**○ History of maternal oligohydramnios
○ Bilious vomiting
○ Scaphoid abdomen
○ Sage of meconium during first 24 hours
○ Gush of offensive diarrhea
○ I don't know

**Intussusception is characterized by:**○ Peak incidence between 10-15 years
○ Ten percent (10%) of cases are idiopathic
○ It is an acute surgical emergency that needs rapid intervention
○ Surgical operation is required for all cases
○ Non-surgical reduction is indicated for complicated cases
○ I don't know

**Which of the following is true regarding testicular torsion:**○ It refers to twisting of the spermatic cord structures causing strangulation of testicular blood supply
○ The patient should be observed and investigated for few days before the operation to exclude epididymo-orchitis
○ It is characterized by chronic testicular pain
○ Doppler ultrasonography is not helpful in diagnosis
○ Surgical intervention is on elective basis
○ I don't know

**Plastic Surgery department**

**Superficial burns are considered which degree:**○ Third
○ Second
○ First
○ Fourth
○ I don't know

**Tissue composition flaps do not include:**○ Skin
○ Fascia
○ Bone
○ Vessels
○ I don't know

**The following is hypothenar muscle of the hand:**○ Abductor digiti minimi
○ Opponens pollicis
○ Abductor Pollicis brevis
○ Flexor Pollicis brevis
○ I don't know

**What is the most common maxillofacial trauma:**○ Mandibular fracture
○ Nasal fracture
○ Frontal bone fracture
○ Orbitozygomatic fracture
○ I don't know

**Toxicology department**

**The commonest complication of prolonged desferroxamine therapy in iron poisoning is:**○ Renal failure
○ Metabolic acidosis
○ Pulmonary oedema
○ I don't know

**A poisoned patient presented to ER with dilated pupils, tachycardia, hyperthermia with dry skin. These manifestations most likely represent which one of the following toxidromes:**○ Opioid toxidrome
○ Anticholinergic toxidrome
○ Sympathomimetic toxidrome
○ I don't know

**N-acetyl cysteine is a specific antidote of one of the following poisons:**○ Salicylates
○ Paracetamol
○ Tricyclic antidepressants
○ I don't know

**Indications for antibiotics in cases of hydrocarbon ingestion include:**○ WBCs: 12×10³/microL
○ Associated vomiting
○ Respiratory distress
○ I don't know

**Effective first aid for snake poisoning is:**○ Tourniquet
○ Assurance
○ Incision and suction
○ I don't know

**Emergency Hospital department**

**A man came to the Emergency department with a cut wound in his forehead caused by a glass bottle:**

**A- Which suture material you should use?**○ Prolene 4-0
○ Vicryl
○ Prolene 6-0
○ I don't know

**B- What's your next step?**○ Let him leave.
○ Write a prescription of medicines and advise him to take a tetanus prophylaxis.
○ Give him a tetanus prophylaxis then write a prescription.
○ I don't know

**According to Glasgow Coma Scale (GCS), if the patient has: Eye opening to sound, Inappropriate words, and Withdrawal from pain, What's his GCS?**○ 8
○ 10
○ 12
○ I don't know

**In Random Blood Glucose, you consider the patient is diabetic when the RBS is above:**○ 120
○ 165
○ 200
○ 225
○ I don't know

**Neurosurgery department**

**Subdural hematoma most commonly results from ?**○ Injury to cortical bridging veins
○ I don't know
○ Rupture of intracranial aneurysm
○ Rupture of cerebral AVM
○ Hemophilia

**Most common cause of subarachnoid hemorrhage is ?**○ Bleeding disorders
○ Arterio-venous malformations
○ I don't know
○ Aneurysm
○ Hypertension

**The most common source of bleeding in extradural hematomas is:**○ I don't know
○ Bridging veins
○ Middle meningeal artery
○ Middle cerebral artery
○ Skull fractures

**What is the most common posterior fossa tumor in pediatrics?**○ Medulloblastoma
○ Ependymoma
○ Astrocytoma
○ Hemangioblastoma
○ I don't know

**What is the most common level of lumbar disc prolapse?**○ L1-L2
○ L2-L3
○ L3-L4
○ L4-L5
○ L5-S1
○ I don't know

**Vascular Surgery department**

**Best investigation for varicose veins:**○ X-Ray
○ Duplex
○ MRI
○ I don't know

**Hemangioma best treated with:**○ Laser
○ Excision
○ Injection
○ I don't know

**Most common site of venous ulcer:**○ Lateral malleolus
○ Medial malleolus
○ Thigh
○ I don't know

**AV fistula was created and patient was started on hemodialysis. Routine assessment for the patient's AV fistula include:**○ Check the blood pressure every 2H on the affected arm to ensure good circulation
○ Check the skin temperature and pulses proximal to the fistula to assess circulation
○ Palpate the access site for a thrill to assess circulation
○ I don't know

**Most common predisposing factor for lymphedema:**○ Radical mastectomy
○ Hypertension
○ Obesity
○ I don't know

**Radiology department**

**In case of abdominal trauma, the initial radiological assessment is:**○ I don't know
○ Abdominal CT
○ Abdominal MRI
○ Abdominal radiograph
○ FAST

**What is the initial radiological assessment in case of head trauma ?**○ CT brain angiography
○ Non-contrast CT brain
○ Skull radiograph
○ Non-contrast MRI brain
○ I don't know

**This radiological picture is:**

**
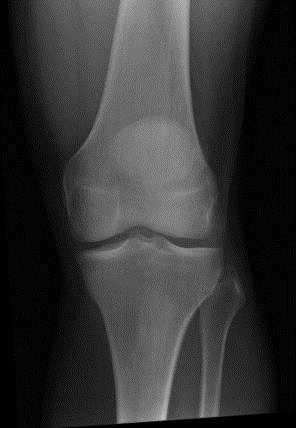
**○ CT knee
○ Knee Sonography
○ Knee MRI
○ I don't know
○ Knee radiograph

**Patient came with manifestations of cerebral stroke, what is the initial radiological assessment?**○ Non-contrast CT brain
○ Skull radiograph
○ Non-contrast MRI brain
○ CT brain angiography
○ I don't know

**This radiological picture is:**

**
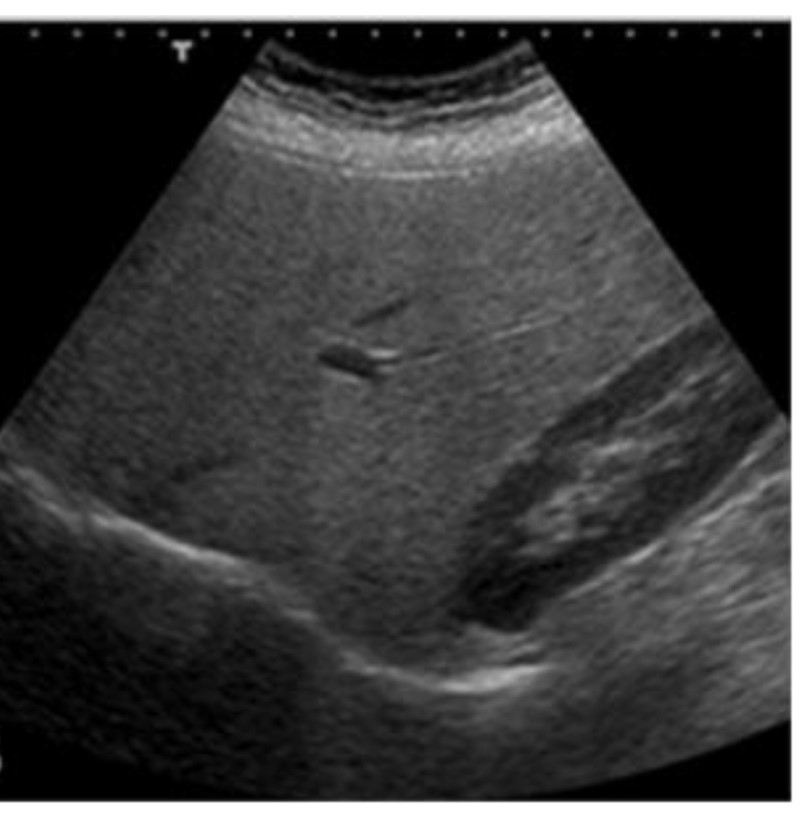
**○ Abdominal radiograph
○ MRI abdomen
○ I don't know
○ CT abdomen
○ Sonography abdomen

**Anesthesia and Surgical ICU department**

**Bupivacaine as one of local anesthetic drugs can be given via following routes except:**○ Spinal
○ IV
○ Supraclavicular block
○ Epidural
○ Local infiltration
○ I don't know

**Safest analgesic drug in CKD patient is:**○ Morphine
○ Ketorolac
○ Paracetamol
○ Pethidine
○ I don't know

**Induction of anesthesia in pediatrics can be:**○ IV
○ IM
○ Inhalational
○ All of the above
○ I don't know

**Ophthalmology department**

**A mother coming to your clinic with her 5-year-old child complaining that her child is always having pain and discomfort in both of his eyes always rubbing his eyes complaining that he feels as if he has a foreign body in his eye associated with redness, and watery secretion of both eyes all symptoms were mainly occurring during summer and spring seasons.**What's your provisional diagnosis?
○ Infection
○ Ocular trauma and foreign body
○ Spring catarrh "Vernal kerato-conjunctivitis"
○ I don't know

**Female patient 60 years old coming to the emergency department complaining of bursting pain in her left eye, on asking about her medical history she mentioned she has been diagnosed with type 2 diabetes mellitus 5 years ago, on examination you notice redness resembling "ciliary injection", so you measure intra-ocular pressure revealing IOP of 70 mmHg.**What is the aim of your immediate management?
○ Treat infection by giving antibiotics.
○ Treating autoimmune attack by giving steroids.
○ Giving drugs to decrease intra-ocular pressure as mannitol.
○ I don't know

**Male patient 17 years old coming to your clinic complaining of pain and redness affecting both eyes, on further questioning he reported suffering from bilateral yellowish white discharge which causes gluing of his lashes on waking up, on examination he had conjunctival injection and the discharge in both eyes.**What is your recommendations to this patient?
○ Hygiene and cold fomentation.
○ Only to use antibiotic eyedrops.
○ Proper hygiene in the form of cleansing his eyes with warm water, using separate towels and bed sheets and to use antibiotic eyedrops in the morning and antibiotic ointment at night for 7-10 days.
○ I don't know

**Male patient 10 years old coming to your clinic complaining of blurring of vision especially while looking at far objects, his mother also reports that he is always squinting his eyes in order to see more clearly.**What is your next step?
○ Reassure and send home.
○ Request ocular CT.
○ Reassure and perform visual acuity testing manually using snellen eye chart and automatically using automatic refractometer.
○ I don't know
